# The Gut Microbiome Is Altered in a Letrozole-Induced Mouse Model of Polycystic Ovary Syndrome

**DOI:** 10.1371/journal.pone.0146509

**Published:** 2016-01-05

**Authors:** Scott T. Kelley, Danalea V. Skarra, Alissa J. Rivera, Varykina G. Thackray

**Affiliations:** 1 Department of Biology, San Diego State University, San Diego, CA, United States of America; 2 Department of Reproductive Medicine, University of California San Diego, La Jolla, CA, United States of America; 3 Center for Reproductive Science and Medicine, University of California San Diego, La Jolla, CA, United States of America; John Hopkins University School of Medicine, UNITED STATES

## Abstract

Women with polycystic ovary syndrome (PCOS) have reproductive and metabolic abnormalities that result in an increased risk of infertility, diabetes and cardiovascular disease. The large intestine contains a complex community of microorganisms (the gut microbiome) that is dysregulated in humans with obesity and type 2 diabetes. Using a letrozole-induced PCOS mouse model, we demonstrated significant diet-independent changes in the gut microbial community, suggesting that gut microbiome dysbiosis may also occur in PCOS women. Letrozole treatment was associated with a time-dependent shift in the gut microbiome and a substantial reduction in overall species and phylogenetic richness. Letrozole treatment also correlated with significant changes in the abundance of specific Bacteroidetes and Firmicutes previously implicated in other mouse models of metabolic disease in a time-dependent manner. Our results suggest that the hyperandrogenemia observed in PCOS may significantly alter the gut microbiome independently of diet.

## Introduction

PCOS is the most common endocrine disorder in women, with an estimated world-wide prevalence of 6–10% based on the NIH criteria and up to 15% using the Rotterdam Consensus criteria [1]. Although the etiology of PCOS remains unclear, the onset of PCOS often occurs in adolescence or the early reproductive years [2, 3]. Women with PCOS often present with hyperandrogenemia, intermittent or absent menstrual cycles, and polycystic ovaries as well as metabolic abnormalities including abdominal adiposity and peripheral insulin resistance. As a result, women with PCOS have an increased risk of infertility, adverse pregnancy outcomes, gestational diabetes, type 2 diabetes, and cardiovascular disease [1, 4–6]. Women diagnosed with PCOS using the NIH criteria of hyperandrogenemia and oligo- or anovulation are at high risk for metabolic dysfunction while women with the normoandrogenic phenotype are not [7]. Given the limitations of current treatments for the metabolic symptoms of PCOS, there is a significant need for studies addressing the etiology and pathology of the PCOS metabolic phenotype.

The large intestine contains a complex community of microorganisms that is important for human health [8, 9]. Studies have demonstrated that the gut microbiome differs in humans with metabolic disorders such as obesity and type 2 diabetes [10–14]. This dysregulation is also observed in mouse models of obesity and metabolic syndrome, such as leptin deficient *ob/ob* mice, mice fed a high fat diet, and toll-like receptor 5 knockout mice [15–20]. Furthermore, studies have shown that transplantation of the obese gut microbiome into germ-free mice results in an obese phenotype [11, 21, 22], indicating that dysregulation of the gut microbiome may play a causative role in the development of metabolic disorders [23]. However, it remains an open question whether the gut microbiome is disturbed in PCOS women and if the PCOS gut microbiome contributes to the development of the PCOS metabolic phenotype.

## Materials and Methods

### PCOS Mouse Model

Three week-old, C57BL/6N female mice (Harlan Laboratories, Indianapolis, IN) were housed in a vivarium for one week under specific pathogen-free conditions with an automatic *12h*:*12h light*/*dark* cycle (*light* period: 06.00–18.00) and *ad libitum* access to water and food (Teklad S-2335 diet, Harlan Laboratories). All animal procedures in this study were approved by the University of California, San Diego Institutional Animal Care and Use Committee (Protocol number S14011). At 4 weeks of age, the mice were implanted subcutaneously with a placebo or 3 mg letrozole pellet (Innovative Research of America, Sarasota, FL) that provided a constant, slow release of letrozole (50 μg/day) (n = 10/group). The mice were weighed weekly. A glucose tolerance test was performed on the mice after 5 weeks of placebo or letrozole treatment. The mice were fasted for 6 hours. Blood glucose was measured using a handheld glucometer (One Touch UltraMini, LifeScan, Inc) just before (time 0) a single intraperitoneal injection of glucose was administered (2 g/kg body weight in sterile saline) and at 15, 30, 45, 60, 90, and 120 minutes post injection. At the end of the experiment, the mice were anesthetized with isoflurane, blood was collected via retro-orbital bleeding, vaginal smears were obtained, parametrial fat pads were dissected and weighed, and the ovaries were dissected, fixed in 4% paraformaldehyde at 4°C overnight and stored in 70% ethanol before processing for histology. Serum testosterone levels were measured using a radioimmunoassay (range 10–800 ng/dL) and 17-β estradiol levels were measured using a mouse ELISA (range 3–300 pg/mL) by the University of Virginia Ligand Core Facility. Vaginal cytology was performed to determine the stage of the estrous cycle. Paraffin-embedded ovaries were sectioned at 10 μm and stained with hematoxylin and eosin. Body composition (fat and lean muscle mass) was determined on a separate cohort of mice (n = 6/group) by dual energy x-ray absorptiometry with a GE Lunar Pixi Densitometer Machine after the mice were fasted for 4 h and euthanized with Fatal-Plus.

### Fecal Sample Collection

Fecal samples were collected from the mice prior to treatment with placebo or letrozole and each week for 5 weeks (120 samples total). Fecal samples were frozen immediately after collection and stored at -80°C. Microbial DNA was extracted from the fecal samples using the MoBio PowerSoil DNA Isolation Kit (MoBio Laboratories, Inc, Carlsbad, CA). Successful DNA isolation was confirmed by measuring DNA concentration using a Nanodrop 2000 (Thermo Fisher Scientific, Waltham, MA).

### Next Generation Sequencing (NGS) of 16S rRNA genes

16S ribosomal RNA genes were amplified by PCR using 16S primers (515F and 806R) that target the V4 hypervariable region [24]. The reverse primers also contained unique 12 base pair Golay barcodes that were incorporated into the PCR amplicons [25]. The resulting amplicons were submitted to The Scripps Research Institute NGS core facility where they were cleaned using DNA Clean & Concentrator^™^-25 columns (Zymo, Irvine, CA), quantitated using a Qubit Fluorometer (Life Technologies, Thermo Fisher Scientific,) and pooled. Pooled PCR products were size selected on a 2% agarose gel (290–350 bp), purified using a Zymoclean^™^ Gel DNA recovery kit and used to prepare sequencing libraries following the recommended Illumina protocol involving end repair, A-tailing and adapter ligation. The DNA library was then size selected on a 2% agarose gel (410–470 bp), cleaned using the Agencourt SPRI system (Beckman Coulter, Inc., Indianapolis, IN) and PCR amplified with HiFi Polymerase (Kapa Biosystems, Wilmington, MA) for 12 cycles. The amplified DNA products were again size selected on a 2% agarose gel and purified using the Zymoclean^™^ Gel DNA recovery kit. The purified DNA library was quantitated, denatured in 0.1 N NaOH and diluted to a final concentration of 5 pM before being loaded onto the Illumina single read flow-cell for sequencing on the Illumina MiSeq system along with 4 pM PhiX control library.

### Analysis of 16S rRNA Bacteria Diversity

The sequences were analyzed using the open source software pipeline Quantitative Insights Into Microbial Ecology (QIIME version 1.8.0–20140103) [26]. The 16S rRNA sequence data was demultiplexed and then quality filtered using the default QIIME parameters using the split_libraries.py script [27]. This resulted in an average of 29,000 sequences per sample with a success rate of 99% (119/120 samples). Sequences were clustered using a denovo OTU picking approach (pick_de_novo_otus.py) with usearch [28]. Sequences were assigned to OTUs with an assumed 97% threshold of pairwise identity for bacterial species by comparison with the Greengenes reference database [29] using the RDP classifier [30]. OTUs present in less than 25% of the samples were discarded from the dataset to minimize the effect of spurious, low abundance sequences using the filter_otus_from_otu_table.py script resulting in 1406 OTUs. Sequences were then aligned using PyNast [26] and a phylogenetic tree constructed using FastTree [31]. Chao1 estimator for species richness (defined as the total number of unique species in an ecosystem) and Faith’s Phylogenetic Diversity (Faith’s PD), which measures the biodiversity of an ecosystem by calculating the total branch lengths on a phylogenetic tree of all members of the ecosystem [32, 33], were calculated using the alpha_diversity.py script. The beta_diversity_through_plots.py script was used to compute weighted and unweighted UniFrac distances. UniFrac measures the similarity among microbial communities by calculating the shared phylogenetic diversity between pairs of microbial communities [34]. The smaller the UniFrac distance between two microbial communities, the more similar the communities are in their overall diversity. The “weighted” UniFrac distance metric incorporates the abundance of specific taxa in each community into the UniFrac distance calculation while “unweighted” UniFrac ignores abundance information. Taxonomic distributions across sample categories were calculated (from phylum to genus) using the summarize_taxa_through_plots.py script. The 16S rRNA sequences were deposited into figshare at http://dx.doi.org/10.6084/m9.figshare.1554900 along with the mapping file at http://dx.doi.org/10.6084/m9.figshare.1554901.

### Statistical Analyses

Two-dimensional PCoA plots were constructed using the make_2d_plots.py script in QIIME. ANOSIM tests using weighted and unweighted UniFrac distances (between treatments and among time points within treatments) were performed using the compare_categories.py script. The make_distance_boxplots.py script was used to create box plots and perform *t*-tests (Bonferroni corrected for multiple comparisons) comparing average UniFrac distances within and among samples collected at different time points. Kruskal-Wallis non-parametric tests (corrected for multiple comparisons via Bonferroni and FDR techniques) were used to determine whether the abundance of OTUs differed among treatments using the group_significance.py script. Pearson product-moment correlation, Spearman’s rank correlation, box plots, 1-factor ANOVAs, and 2-factor repeated measures ANOVAs were performed using the R statistical package (version 2.14.1).

## Results

### Letrozole treatment of peripubertal female mice results in both reproductive and metabolic hallmarks of PCOS

**Fig 1:**
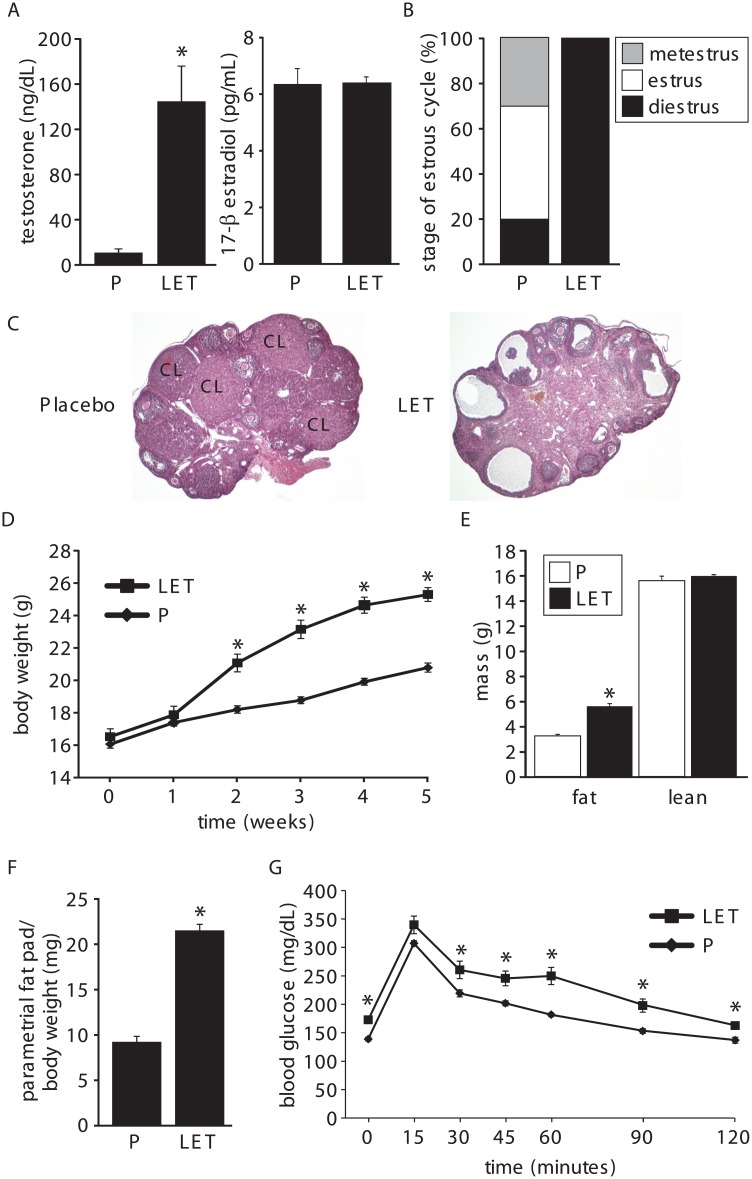
Letrozole treatment of 4-week-old female mice resulted in both reproductive and metabolic hallmarks of PCOS. Five weeks of letrozole treatment resulted in increased serum testosterone (A), acyclicity (B), polycystic ovaries (C), increased body weight (D), increased fat mass (E), increased parametrial fat pad relative to body weight (F), and an impaired glucose tolerance test with elevated basal glucose levels and significant delays in glucose clearance after intraperitoneal injection previously published in [35] (G); n = 10 placebo (P) and letrozole (LET). * p<0.05 Student’s *t*-test. CL, corpus luteum.

We employed a letrozole-induced PCOS mouse model to determine whether PCOS is associated with an altered gut microbiome. In this model, letrozole, a nonsteroidal aromatase inhibitor, was used to increase endogenous testosterone levels by limiting the conversion of testosterone to estrogen. In our study, 4 week-old female C56BL/6 mice were implanted with a subcutaneous pellet and exposed to placebo or 50 μg/day of letrozole for 5 weeks. Similarly to a recent report [35], 5 weeks of 50 μg/day letrozole treatment resulted in hallmarks of PCOS including elevated testosterone, acyclicity and polycystic ovaries (Fig 1A–1C). Circulating levels of 17-β estradiol were not significantly altered after 5 weeks of letrozole treatment and were in the diestrus range similar to follicular-phase levels observed in PCOS women (Fig 1A). The letrozole mouse model also had multiple metabolic features of PCOS including obesity, increased fat mass, increased abdominal (parametrial) fat, elevated basal glucose levels, and impaired glucose tolerance (Fig 1D–1G).

### Letrozole treatment decreases the species abundance and phylogenetic diversity of the gut microbiome

**Fig 2:**
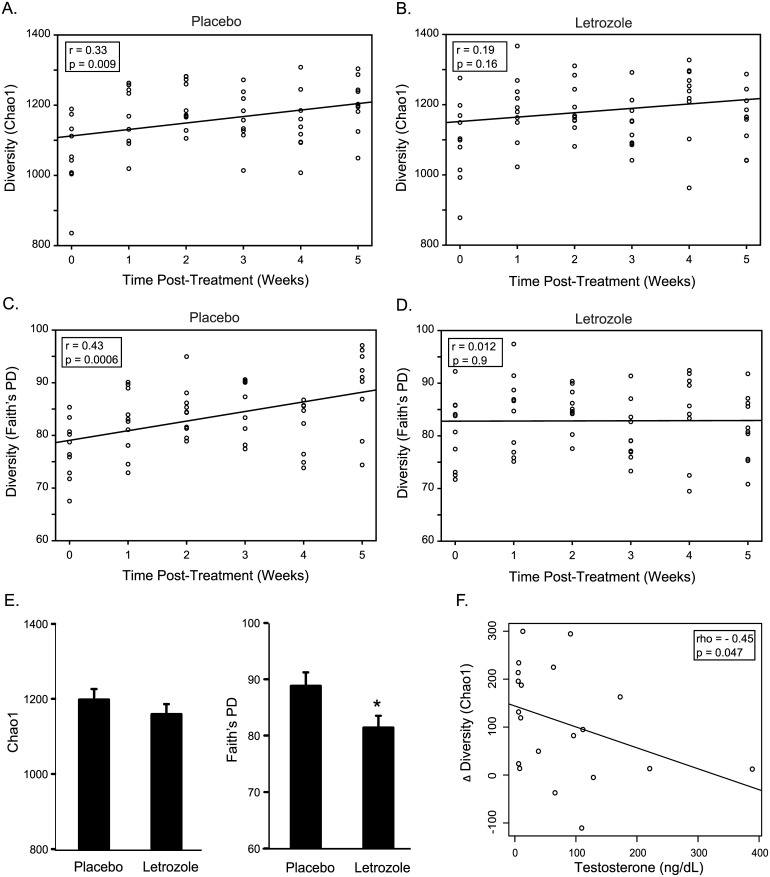
Letrozole treatment blocks the increase in species abundance and phylogenetic diversity observed over time. Chao1 species (OTU) richness estimates per sample at each collection time for placebo (A) and letrozole-treated mice (B). Faith’s phylogenetic diversity estimate per sample at each collection time for placebo (C) and letrozole-treated mice (D). Results of Pearson’s correlation shown in box inset along with line of best fit. Comparison of Chao1 and Faith’s PD after 5 weeks of placebo or letrozole treatment (ANOVA; * p<0.05) (E), Scatter plot and trend line showing relationship between testosterone levels and change in Chao1 diversity within individuals (time 5 minus time 0) (F). Result of Spearman’s correlation shown in box inset.

We investigated how letrozole treatment altered the overall species richness and phylogenetic diversity of the gut microbiome using the Chao1 estimator for species richness and Faith’s PD [32, 33]. Placebo-treated mice showed a highly significant positive correlation between operational taxonomic unit (OTU) richness and diversity of their gut communities over time as assessed by Chao1 (Pearson’s: r = 0.33; p = 0.009) and Faith’s PD (Pearson’s: r = 0.43; p = 0.0006) (Fig 2A and 2C). Strikingly, letrozole treatment abrogated the increase in gut microbiome diversity observed over time in the placebo-treated mice (Fig 2B and 2D). Direct comparison of placebo and letrozole-treated mice found a significant decrease in alpha diversity after 5 weeks of letrozole treatment (ANOVA: Faith’s PD, p = 0.03; Chao1, p = 0.15; Fig 2E). Moreover, testosterone levels were inversely correlated with alpha diversity (Spearman’s rank correlation: Chao1 rho = -0.45, p = 0.047; Faith’s PD rho = -0.43, p = 0.068; Fig 2F), suggesting that hyperandrogenemia in the PCOS mouse model results in decreased abundance of bacterial species in the large intestine.

### Letrozole treatment changes the beta diversity of the gut microbiome

**Fig 3:**
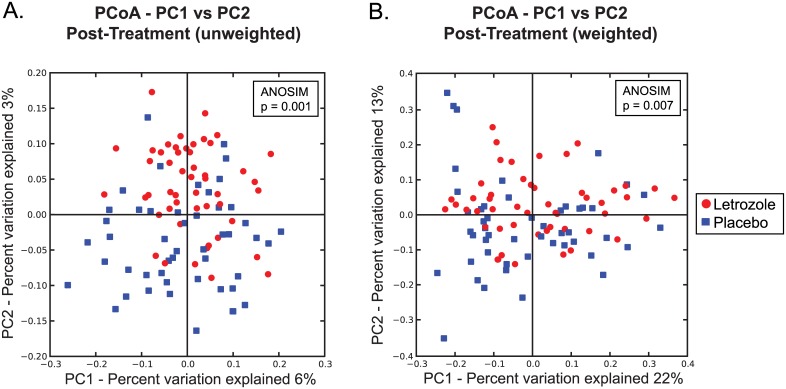
Letrozole treatment results in a significant shift in gut microbiome beta diversity. Principle Coordinates Analysis based on unweighted (A) and weighted (B) UniFrac distances. Comparison of samples collected post-treatment from placebo and letrozole treated mice.

In addition to assessing alpha diversity, we used unweighted and weighted UniFrac analyses to compare the similarity among gut microbial communities (beta diversity) in placebo versus letrozole-treated samples. While Principle Coordinate Analysis (PCoA) plots of the unweighted and weighted UniFrac distances did not completely discriminate between post-treatment placebo and letrozole samples (Fig 3A and 3B), non-parametric Analysis of Similarities (ANOSIM) tests demonstrated that there is a significant difference in the overall bacterial community composition of the gut microbiome between placebo and letrozole-treated mice (ANOSIM: unweighted UniFrac, p = 0.001; weighted UniFrac, p = 0.007).

### Letrozole results in a shift in the gut microbial community after one week of treatment

**Fig 4:**
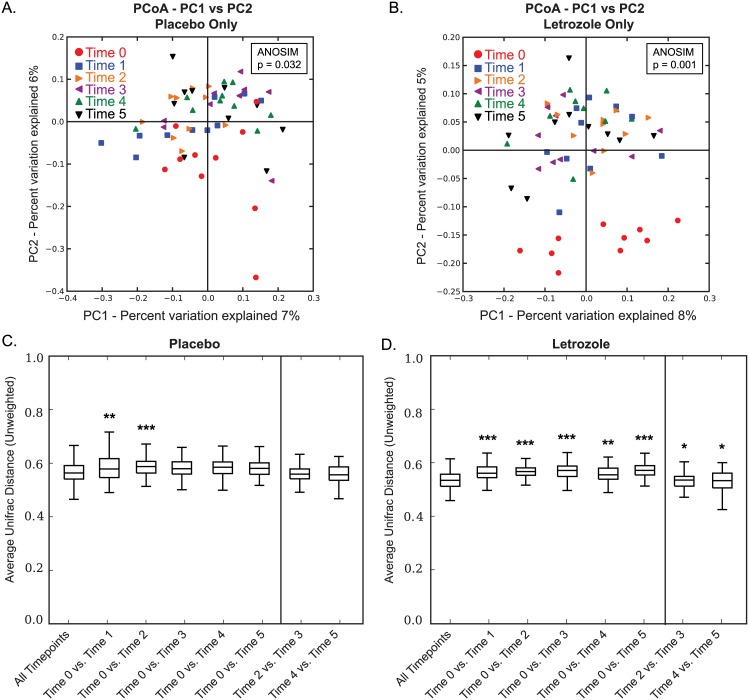
Letrozole results in a shift in beta diversity after one week of treatment. Principle Coordinates Analysis based on unweighted UniFrac distances. Comparison of samples from placebo-treated mice colored by collection time, week 0–5 (A). Comparison of samples from letrozole-treated mice colored by collection time, week 0–5 (B). Box and whisker plots showing mean and variance of average pair-wise unweighted UniFrac distances between collection time (week) 0 and all other collection time points in the study for placebo (C) and letrozole-treated mice (D). Bars above the graphs indicate when mean differences between time points were significantly different from mean UniFrac distances within all time points, * p<0.05, ** p<0.01, *** p<0.001.

We then asked when (i.e., at what time point in the study) did the shift in the gut microbiome occur in the letrozole-treated mice. Interestingly, PCoA plots of the unweighted UniFrac analysis over 5 weeks showed significant differences among time points in both placebo-treated (Fig 4A; ANOSIM: p = 0.032) and letrozole-treated mice (Fig 4B; ANOSIM: p = 0.001), with a clearly visible separation between pre-treatment (time 0) and all post-treatment letrozole samples (Fig 4B). PCoA plots of the weighted UniFrac distances for gut microbial community samples over 5 weeks treatment also found a subtle but significant difference in the gut microbiome over time in the placebo (S1A Fig; ANOSIM: p = 0.002) and after letrozole treatment (S1B Fig; ANOSIM: p = 0.019).

We used *t*-tests corrected for multiple comparisons to ask whether the average UniFrac distance between samples at any given pair of time points (e.g., placebo time 0 vs. placebo time 1 samples) were significantly different than the average UniFrac distance among the samples collected at all time points. The box plots in Fig 4C show that, for placebo treated mice, we observed significant increases in the average unweighted UniFrac distance between samples obtained prior to treatment (week 0) versus samples collected in the following two weeks (times 1 and 2). In contrast, we found a significant increase in the average UniFrac distance between the samples collected at week 0 compared to those collected during all subsequent weeks (times 1–5) in the letrozole-treated mice (Fig 4D). While Time 2 vs. Time 3 and Time 4 vs. Time 5 were significantly different in the letrozole-treated mice, the other pair-wise combinations were not significantly different compared with the average distance among all time points (Fig 4C and 4D). Comparison of the average weighted UniFrac distance between samples demonstrated a significant increase between samples collected at week 0 compared to those at 1, 3, 4 and 5 weeks (S1C Fig). In the letrozole-treated mice, we found a significant increase in the average UniFrac distance between the samples collected at week 0 compared to those collected between weeks 2–5 weeks. (S1D Fig).

### Letrozole treatment alters the abundance of multiple Bacteroidetes and Firmicutes in the gut microbiome

**Fig 5:**
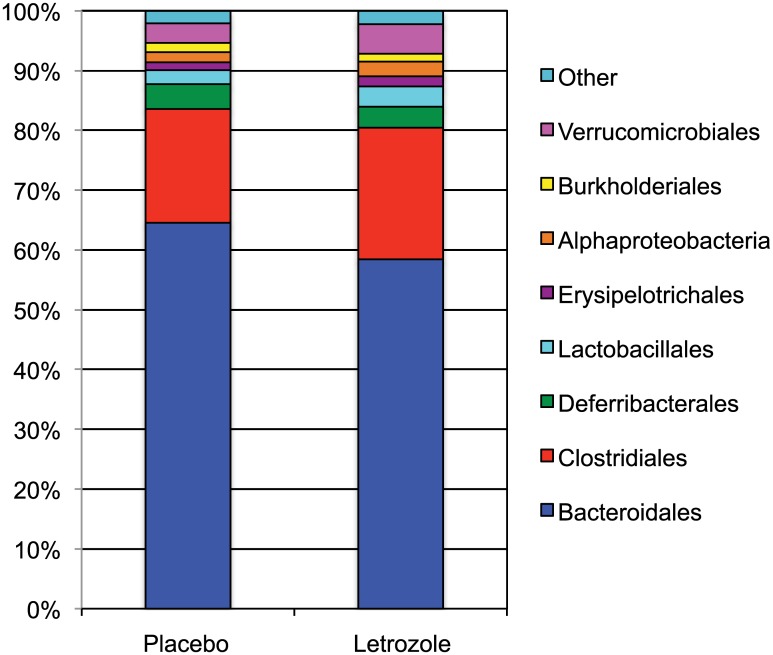
Letrozole treatment resulted in a decrease in Bacteroidales and increase in Clostridiales. Relative abundance of specific bacteria grouped by order in placebo and letrozole treated mice at the end of the study (week 5).

Having detected a significant letrozole-induced change in the gut microbial community, we then investigated which members of the gut community changed after letrozole treatment. In all samples, the majority of OTUs (~85–90%) belonged to two bacterial phyla: Bacteroidetes and Firmicutes. While there was no change between placebo and letrozole treatment at the phyla level, letrozole treatment resulted in a discernable decrease in Bacteroidales and an increase in Clostridiales when grouped by order (Fig 5). We then determined whether the mean relative abundances of specific bacterial taxa were significantly different in the gut microbiome of letrozole-treated mice compared to placebo-treated mice using a non-parametric Kruskal-Wallis test (Bonferroni and False Discovery Rate (FDR) corrected for multiple comparisons). The relative abundance of 48 different OTUs changed significantly with letrozole treatment (S2 Fig). Except for two unclassified bacterial species and one Actinobacteria (genus *Bifidobacterium*), the other 45 OTUs were Bacteroidetes and Firmicutes (28 Bacteroidales, 16 Clostridiales and one Erysipelotrichales). Letrozole treatment resulted in a relative decrease in all of the Bacteroidales OTUs including 21 uncultured OTUs identified as S24-7 and 1 in the family Rikenellaceae, genus *Alistipes*. Letrozole treatment resulted in an increase in a majority of the Firmicutes OTUs including 5 OTUs identified as Lachnospiraceae, one in the family Erysipelotrichaceae, genus *Allobaculum* and two in the family Ruminococcaceae. Only 4 Firmicutes OTUs significantly decreased after letrozole treatment (two Clostridiales, one Ruminococcaceae and one Dehalobacteriaceae).

**Fig 6:**
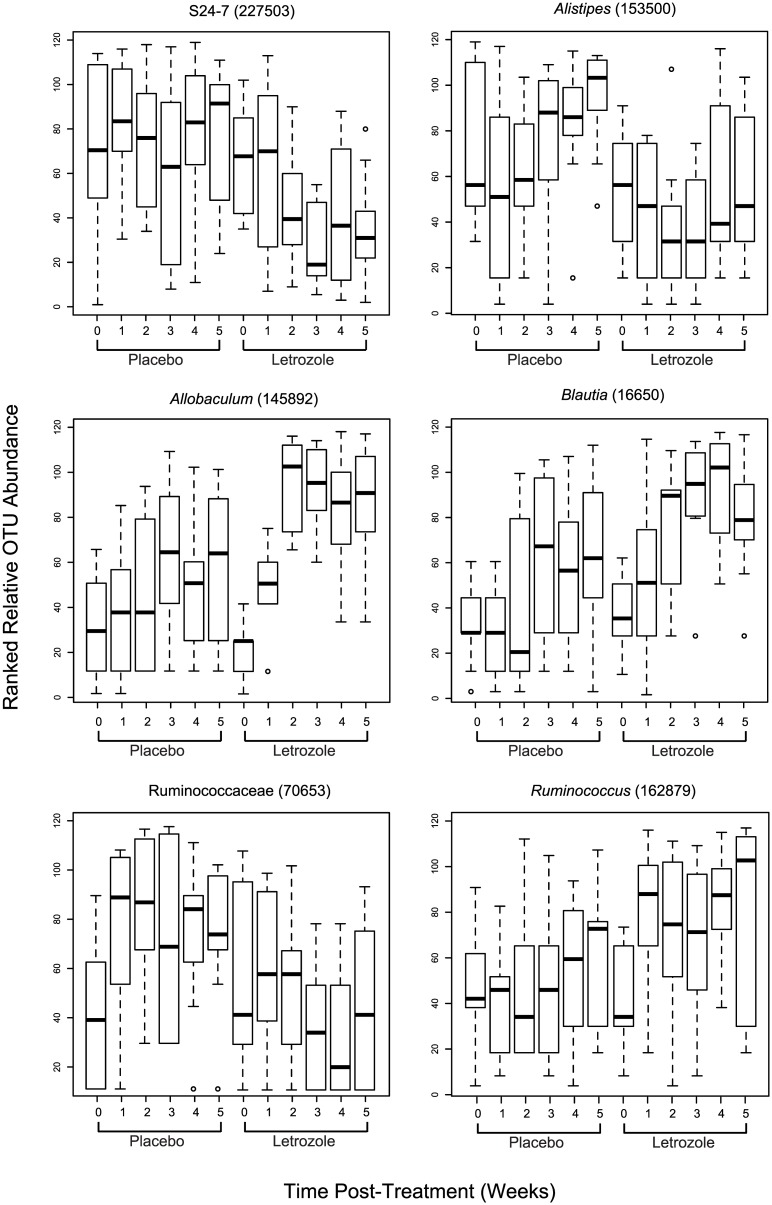
Time-dependent response to letrozole treatment varies among bacterial species. Box and whisker plots showing average ranked abundances of 6 OTUs at each collection time for placebo and letrozole treated mice, as indicated.

Finally, we used box plots to compare and contrast the time-dependent changes in the relative abundance of several Bacteroidetes and Firmicutes OTUs that significantly differed between treatments. The relative abundance of OTU 227503 identified as S24-7 bacteria remained constant during the 5 weeks of the study in the placebo-treated mice, while there was a marked decrease by the second week of letrozole treatment that continued to the end of the experiment (Fig 6). A similar trend occurred in the other S24-7 OTUs (data not shown). In comparison, the relative abundance of the *Alistipes*-identified OTU increased dramatically after 3 weeks of the study in the placebo mice, while this same OTU decreased after two weeks of letrozole treatment (Fig 6). In contrast to the Bacteroidetes OTUs, which consistently showed a rapid decline in abundance post-letrozole treatment while remaining stable or slightly increasing in the placebo-treated animals, the response of the Firmicutes was more OTU specific. For instance, the Firmicutes OTUs in the genera *Allobaculum*, *Blautia* and *Ruminococcus* increased dramatically after letrozole treatment while experiencing little or modest increases in relative abundance over the 5 weeks of placebo treatment (Fig 6). In contrast, one of the Firmicutes OTUs belonging to the Ruminococcaceae family increased dramatically after one week before stabilizing in placebo-treated mice, while the same OTU was little changed after letrozole treatment (Fig 6). Two-factor (treatment x time) repeated measures ANOVAs found significant effects of time or significant interactions between treatment and time for all 6 OTUs.

## Discussion

Our results demonstrated that there are significant changes in the composition of the gut microbiome in a letrozole-induced mouse model of PCOS. Letrozole treatment of peripubertal, 4-week-old female mice resulted in a clear time-dependent shift in the overall gut microbial community, as well as significant reductions in its overall species richness and phylogenetic diversity. Moreover, letrozole treatment resulted in dramatic time-dependent shifts in the relative abundance of specific bacterial OTUs, most of them members of the Bacteroidetes and Firmicutes that have been previously reported to be altered in other mouse models of metabolic disease. Notably, these changes occurred after treatment with letrozole, an aromatase inhibitor that induces hyperandrogenemia by reducing the conversion of testosterone to estrogen.

One possible explanation for the changes we observed in the gut microbiome is that letrozole had a direct effect on the gut microbiome. However, this seems unlikely due to the fact that the aromatase gene evolved in chordates and that aromatase activity has not been described in bacteria. It is more likely that letrozole effects on host steroid hormone levels resulted in changes in the gut microbiome and host metabolism although it remains to be determined whether alterations in the gut microbiome cause the PCOS metabolic phenotype or are a result of the PCOS metabolic phenotype. Future studies employing fecal transplantation of the PCOS gut microbiome into germ-free mice will help address this question. Though many studies have used feces as a proxy for the gut microbiome, it should be noted that feces may not accurately reflect changes in the gut microbiome in the PCOS mouse model. Future sampling of the large intestine including bacteria associated with the gut epithelium as well as bacteria in the lumen will help address whether changes in the fecal microbiome correlate with changes in the gut.

We observed a highly significant difference in the species richness of the gut microbiome between placebo and letrozole-treated mice over the course of the study. Specifically, there was a strong positive correlation between bacterial species richness and time in placebo-treated mice and no correlation in letrozole-treated mice (Fig 2A and 2B). A similar, even more robust, pattern was observed using Faith’s PD estimate (Fig 2C and 2D), indicating that the changes in species richness was spread across phylogenetically diverse groups of bacteria. Direct comparison of placebo and letrozole treated mice demonstrated a significant decrease in phylogenetic diversity after 5 weeks of letrozole treatment while the decrease in species richness was not statistically significant, likely due to the relatively small number of mice per group. Generally speaking, higher overall biodiversity is known to improve ecosystem function and productivity in macrobiotic systems [36]. More recently, the species richness of the gut microbial ecosystem has also been correlated with human health [37–39].

As illustrated in Figs 2 and 3, there was considerable variability in the composition of the gut microbiome among individual mice. Given the inbred nature of the C57BL/6 mouse strain, this is likely due to environmental factors. Despite the high degree of individual variance and overlap in the gut microbiome, PCoA of the UniFrac distances indicated that there was a significant shift in the gut microbiome post-treatment between placebo and letrozole-treated mice (Fig 3). PCoA of the unweighted UniFrac distances in placebo and letrozole-treated mice showed a discernable difference in the gut microbiome samples from the start of the experiment (Time 0) compared to post-treatment (Time 1–5) samples (Fig 4A and 4B) although the separation was more distinct for the letrozole-treated animals. Comparison of the average unweighted UniFrac distance over time confirmed that the shift in beta diversity from pre-treatment (Time 0) to the first week clearly occurred in both placebo and letrozole-treated mice (Fig 4C and 4D). Differences in the unweighted and weighted UniFrac results (S1 Fig) indicate that letrozole treatment may not only alter abundance of taxa, but may also significantly shift the underlying phylogenetic diversity. These results suggest that letrozole treatment changes the complexity and composition of the gut microbiome, possibly affecting its normal functioning, and that the effects of letrozole during puberty may, in turn, influence the gut microbial community in adulthood. In future studies, it will be interesting to determine if letrozole treatment administered in adulthood has the same effects on the gut microbiome as during puberty. Given that the effects of letrozole treatment on fertility appears to be reversible [35], future research should determine whether the effects of letrozole on the gut microbiome are similarly reversible or whether letrozole treatment results in permanent disruption of the gut microbiome.

There are several potential explanations for the shift in the beta diversity of the gut microbial community in the placebo-treated mice after the first week of the experiment. Since these mice were weaned at ~3 weeks, it is possible that the change in the gut microbiome reflects the change from a liquid milk diet to a solid diet. Alternatively, this shift in the microbiome may reflect the developmental stage of the mice. Several studies have observed shifts in the gut microbiome during mouse development [40, 41] but it is not clear whether changes in the gut microbial community after 4 weeks of age result from aging or from developmental changes associated with puberty. The fact that puberty is a hormone-driven event and the discovery that letrozole treatment results in significant effects on the gut microbiome during this time, suggest that puberty may play an important role in shaping the mammalian gut microbiome. Interestingly, the shift in the microbiome of the letrozole-treated mice after one week of treatment is followed by significant weight gain in the second week of the experiment (Fig 1), suggesting that changes in gut microbiome may result in increased adiposity. It is unknown at this time whether this shift precedes other metabolic disturbances, such as glucose intolerance or insulin resistance.

Consistent with previous studies of the mouse gut microbiome, members of the phyla Bacteroidetes and Firmicutes comprised the majority of the mouse gut bacterial diversity, with the Bacteroidetes consistently the most numerous across all time points in both treatment groups. (Interestingly, although several studies have found Bacteroidetes to predominate over Firmicutes as ours did [42–44], others have found the reverse pattern [15, 17, 45].) While we did not observe a change in the relative abundance of bacterial phyla between placebo and letrozole treatment, a discernable difference was observed when the OTUs were grouped by order, with a decrease in Bacteroidales and an increase in Clostridiales (Fig 5). A closer examination of specific bacterial abundances identified 48 different OTUs that differed significantly between treatments (S2 Fig). Analysis of some of these OTUs revealed time-dependent, and often OTU-specific, shifts in mean relative abundance. For instance, in placebo-treated mice, the abundance of the OTUs identified as Bacteroidetes S24-7 remained steady throughout the study, while the abundance of these same OTUs declined markedly after 1 week of treatment in letrozole mice (Fig 6). The Bacteroidetes OTU identified as belonging to the genus *Alistipes* also decreased in the letrozole-treated mice, while increasing sharply in the placebo-treated mice (Fig 6). While the temporal abundance patterns were similar among the Bacteroidetes, the patterns of abundances among the Firmicutes were more variable and sometimes completely opposite one another. For example, one of the Ruminococcaceae-related OTUs increased in abundance in the placebo group and decreased in letrozole-treated animals, while *Allobaculum*, *Blautia* and other Ruminococcaceae-related OTUs did the opposite (Fig 6). These findings of time-dependent and species (OTU)-specific abundance shifts make clear the importance of investigating the effects of gut disturbance at the species level and at multiple time points. They also may help explain why ratios of phyla abundance may not be particularly meaningful measures of gut microbiome composition. Moreover, our data suggest that comparative genomics of reconstructed uncultured genomes (via metagenomics or single cell sequencing) may be useful for understanding differential patterns of abundance shifts in related taxa. Furthermore, studies employing quantitative PCR to measure the change in absolute abundance of specific bacterial species will be useful to corroborate the effects of letrozole on the gut microbiome.

Many of the OTUs in our study that differed significantly between placebo and letrozole treatment have also been shown to be altered in the gut microbiome of diet-induced obesity mouse models. For instance, the relative abundance of Bacteroidetes from the S24-7 family decreased in mice fed a high fat diet [46–48] and after letrozole treatment. Moreover, Ruminococcaceae abundance increased in mice fed a high fat diet [48–51], consistent with what we observed in two Ruminococcaceae OTUs after letrozole treatment. On the other hand, *Alistipes* abundance increased in mice fed a high fat diet [48–51], which is the opposite of what we observed after letrozole treatment. *Allobaculum* and members of the Lachnospiraceae, including the genera *Blautia*, have been reported to either increase or decrease after high fat diet depending on the study [16, 45, 48–50, 52], while we observed a decrease after letrozole treatment. It is important to note that changes in the gut microbiome in the letrozole-induced PCOS mouse model occurred in the absence of diet manipulation, while studies of diet induced obese mice are confounded by the effect of an altered diet. Taken together, these comparisons suggest that letrozole treatment impacts a common suite of bacteria implicated in metabolic disease but in a manner specific to the effect of letrozole on endogenous testosterone levels. In future studies, it will be interesting to investigate the functional effect of the altered gut microbiome using metagenomic and metabolomic analyses.

In summary, letrozole treatment of peripubertal female mice decreased mouse gut bacterial diversity and precipitated species-specific and time-dependent shifts in the relative abundance of particular Bacteroidetes and Firmicutes, many of which have been implicated in other mouse models of metabolic disease. The idea that steroid hormones may regulate the gut microbiome is not without precedent, though this is the first study to demonstrate this effect via manipulation of endogenous testosterone levels. The gut microbiome of pregnant women has been reported to shift markedly between the first and third trimester, a time of tremendous hormonal change [53]. Additionally, it has been shown that the development of a diabetic phenotype in a non-obese diabetic mouse model depends on the steroid hormone status of the animals. Female mice develop diabetes while male mice are resistant unless they are castrated, and female mice treated with testosterone or the gut microbiome from male mice are protected from developing diabetes [40, 41]. Thus, our study provides support for the idea that steroid hormone levels may regulate composition of the gut microbial community and metabolism. Our observation of gut microbiome alteration in a letrozole-induced PCOS mouse model suggests that a “dysbiosis” or microbial imbalance in the gut microbiome may also occur in women with PCOS. This discovery also creates opportunities for future mechanistic studies investigating the role of the gut microbiome in the PCOS metabolic phenotype, as well as a means to assess the efficacy of current and potential treatments such as insulin sensitizers, anti-androgens, estrogens, or pre/probiotics on the gut microbiome and the PCOS metabolic phenotype.

## Supporting Information

S1 Fig Principle Coordinates Analysis based on weighted UniFrac distances. Samples from placebo-treated mice colored by collection time, week 0–5 (A). Samples from letrozole-treated mice colored by collection time, week 0–5 (B). Box and whisker plots showing mean and variance of average pair-wise weighted UniFrac distances between collection time (week) 0 and all other collection time points in the study for placebo (C) and letrozole-treated mice (D). Bars above the graphs indicate when mean differences between time points were significantly different from mean UniFrac distances within all time points, * p<0.05, ** p<0.01, *** p<0.001.
(EPS)

S2 Fig Letrozole treatment results in significant changes in 48 bacterial OTUs as assessed by Kruskal-Wallis test. The denovo OTU identifier, Test-Statistic, uncorrected p-value, corrected p-value using FDR and Bonferroni correction for multiple comparisons, placebo mean of ranked relative abundance over the entire study (weeks 0–5), letrozole mean from weeks 0–5, and the taxonomy of the closest match as identified using Greengenes are shown.
(EPS)
